# The PROTAC technology in drug development

**DOI:** 10.1002/cbf.3369

**Published:** 2019-01-02

**Authors:** Yutian Zou, Danhui Ma, Yinyin Wang

**Affiliations:** ^1^ The State Laboratory of Membrane Biology, Department of Basic Medicine, School of Medicine Tsinghua University Beijing China; ^2^ Department of Science Brookwood High School Snellville Georgia

**Keywords:** cancer, drug development, PROTAC, protein degradation, small molecule

## Abstract

Currently, a new technology termed PROTAC, proteolysis targeting chimera, has been developed for inducing the protein degradation by a targeting molecule. This technology takes advantage of a moiety of targeted protein and a moiety of recognizing E3 ubiquitin ligase and produces a hybrid molecule to specifically knock down a targeted protein. During the first decade, three pedigreed groups worked on the development of this technology. To date, this technology has been extended by different groups, aiming to develop new drugs against different diseases including cancers. This review summarizes the contributions of the groups for the development of PROTAC.

**Significance of the study:**

This review summarized the development of the PROTAC technology for readers and also presented the author's opinions on the application of the technology in tumor therapy.

## 1 INTRODUCTION

Strategies on cancer therapy using drugs include antibodies, siRNAs, and small molecules to block the activity of oncogenic proteins. Antibodies are of very potent specificity but remains difficult in cell permeability. Inhibition of gene expression by using siRNAs was exciting, but difficulty of the delivery system and the problem of off‐target impeded its application. Conventionally, small chemical molecules were extensively screened and synthesized to bind specific proteins, aiming at inhibiting the activity of the protein. However, drug resistance occurs when a small‐molecule drug is frequently used, and in some special cases, inhibitors even leads to accumulation of the proteins.^1^ Also, for some of the proteins such as Ras, with a critical mutation during tumourigenesis, many efforts failed to identify small inhibitors because of its undruggable structure. Recently, drug designers attempted to target protein‐protein interaction, which is critical for signalling transduction, to develop small inhibitors. Intriguingly, a great effort has been made to develop new strategies for inducing protein degradation. One of the promising technology is PROTAC, proteolysis targeting chimera.^2^

**Figure 1:**
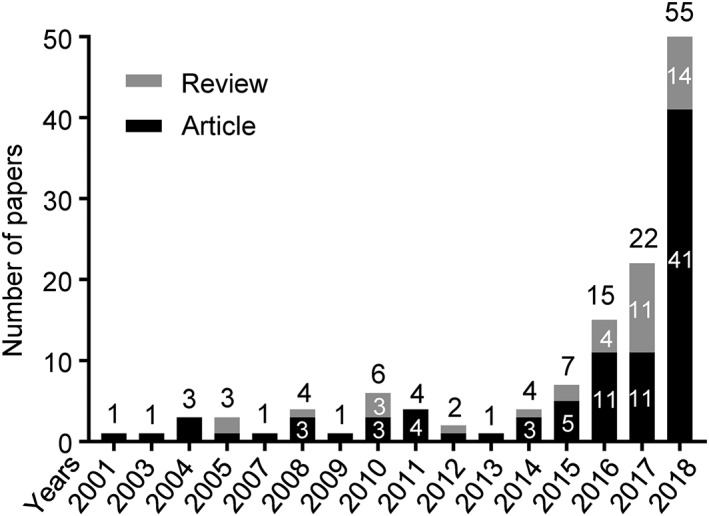
A graph view of the publications on the proteolysis targeting chimera (PROTAC) technology. Research articles and reviews on PROTAC were searched from Pubmed (https://www.ncbi.nlm.nih.gov/pubmed). The literatures were presented chronologically from 2011. Numbers up columns indicate the total number of article and review papers

PROTAC is a strategy that utilizes the ubiquitin‐protease system to target a specific protein and induce its degradation in the cell.^2^ The normal physiological function of the ubiquitin‐protease system is responsible for clearing denatured, mutated, or harmful proteins in cells.^3^, ^4^ PROTAC takes advantage of the cell's own protein destruction mechanism to remove specifically targeted proteins from cells.^5^ To date, the PROTAC technology can be used to target varieties of proteins, including transcription factors, skeleton proteins, enzymes, and regulatory proteins.^6^ Recently, this technology has drawn the great attention of many researchers in different fields from cancer to neuron diseases.^7^ This is mainly due to the potent ability in inducing targeted protein degradation by designed PROTAC molecules. Many studies have showed that degrading a protein is better than inhibiting a protein for the anticancer activities.^8^ From 2001 to 2018, more than 30 review articles and 80 research papers have been published according to Pubmed (Figure 1).^5^, ^8^, ^9^, ^10^, ^11^, ^12^, ^13^, ^14^, ^15^, ^16^, ^17^, ^18^, ^19^, ^20^

## 2 PROTAC'S PREDECESSOR

**Figure 2:**
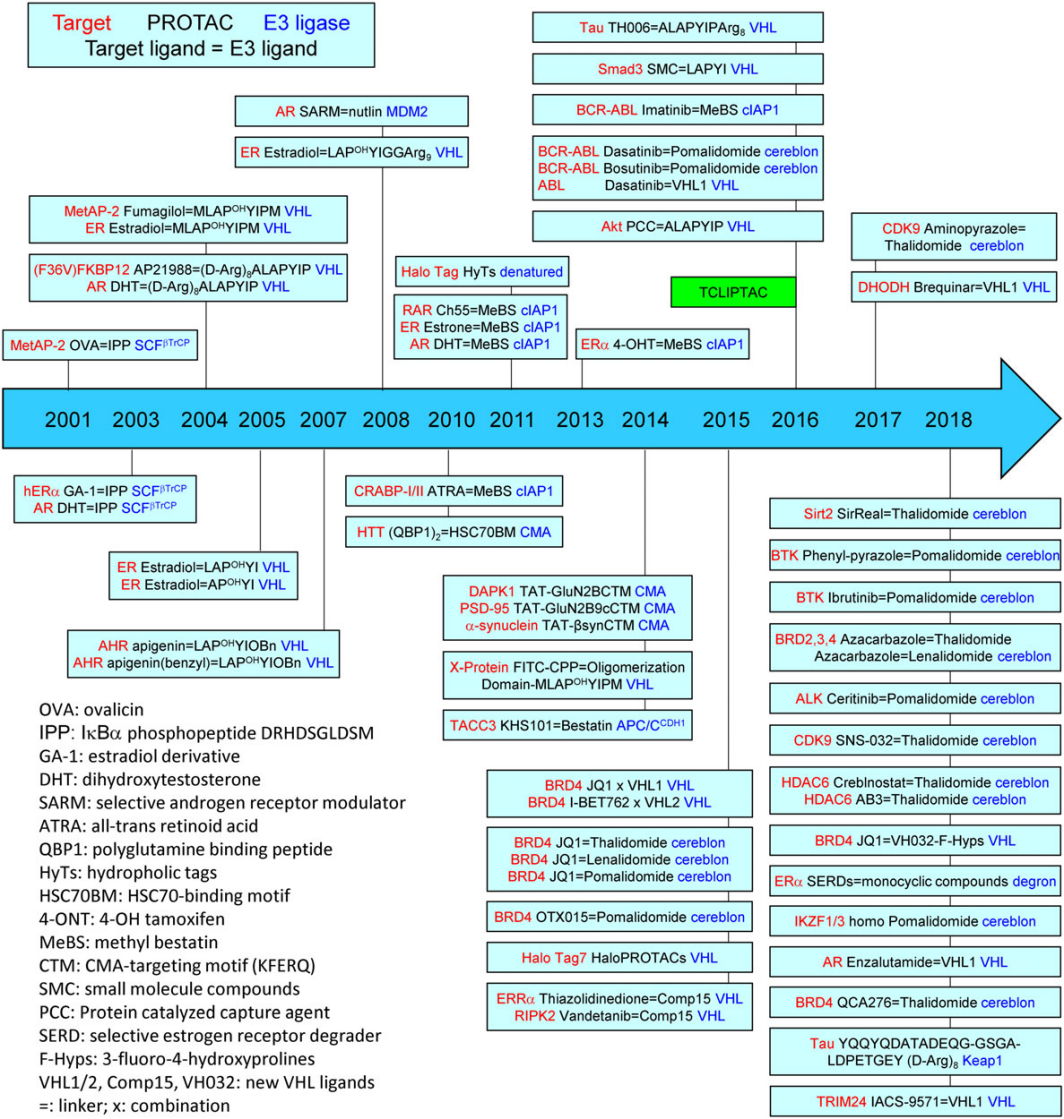
A diagram to demonstrate the proteolysis targeting chimera (PROTAC) molecule designs. Only effective PROTACs are presented. Targeted protein is labelled in red colour, and the recruited E3 ubiquitin ligase is labelled in blue colour. A box indicate a research group. Abbreviations of the ligands are listed

In an attempt to modify the toxicity of geldanamucin, a natural product benzoquinoen ansamycin antibiotic, which binds HSP90, a molecule chaperone for many proteins including estrogen receptor (ER), several groups observed that geldanamycin quickly induced degradation of many proteins including ER, HER‐2, Raf‐1, IGFR1R, mutated v‐Src, Brc‐Abl, and p53. Therefore, a rational strategy for reducing the toxicity of geldanamycin was to link it to estradiol so that it could be able to target ER specifically.^21^ Similarly, geldanamycin was considered to connect to testosterone for targeting androgen receptor (AR).^22^ These studies originally proposed a concept that a hybrid molecule could be able to mediate specific degradation of the targeted proteins.^20^ Alternatively, attempts were made to use chimeric proteins from the SCF proteolytic machinery, a multimeric E3 ubiquitin ligase complex.^23^, ^24^ In 2000, Zhou et al engineered the SCF E3 ubiquitin ligase complex, by using a specific protein interaction domain to target pRb in yeast and human osteosarcoma SARS‐2 cells.^4^ These efforts could be regarded as the predecessor of PROTAC, which was later on developed by Kathleen M. Sakamoto and Raymond J. Deshaires, in collaboration with Kyungbo Kim, Frank Mercurio, and Craig M. Crews in 2001 and 2003.^2^, ^25^ For the first decade from 2001 to 2010, these pedigreed groups led by Raymond J. Desharies, Kathleen M. Sakamoto, Kyungbo Kim, and Craig M. Crews dominantly contributed to the development of this new technology (Figure 2). This review intends to summarize the application of PROTAC since it is developed.

## 3 PEPTIDE‐BASED PROTAC TECHNOLOGY

**Figure 3:**
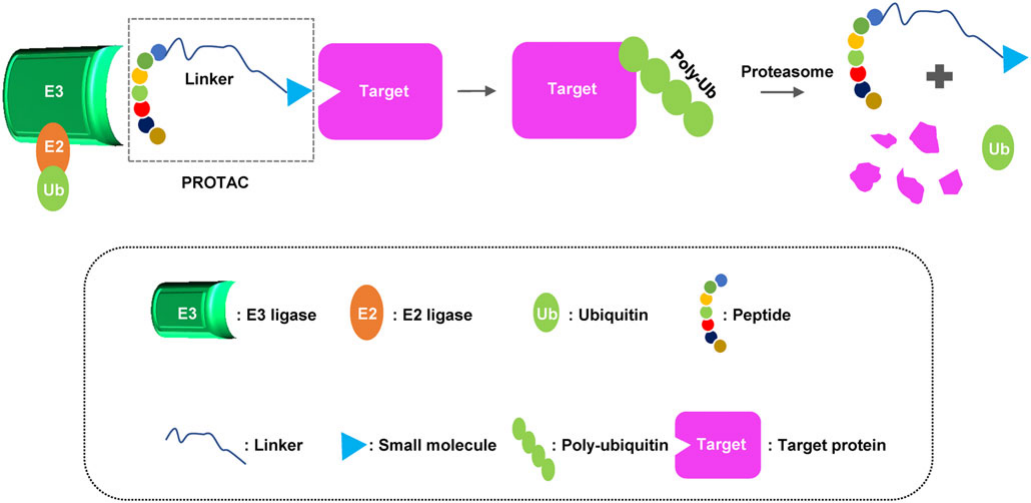
A schematic diagram of a peptide‐based proteolysis targeting chimera (PROTAC). This PROTAC is composed of a short peptide that binds to E3 ubiquitin ligase and a small molecular that binds to target protein, respectively, followed by polyubiquitination and proteasome degradation of target

Kathleen M. Sakamoto reported the first bifunctional or hybrid molecule named PROTAC, which recruits the ubiquitin‐proteasome system, where an E3 ubiquitin ligase is linked to target proteins for degradation.^2^ This collaborative group designed a chimeric molecule based on the angiogenesis inhibitor ovalicin, by linking to the IκB‐α phosphopeptide. Since oyalicin covalently binds MetAP‐2 (methionine aminopeptidase‐2) and the phosphopeptide is recognized by the F‐box, cMetAP‐2 could be targeted by this hybrid molecule that recruits the E3 ubiquitin ligase β‐TRCP. As was expected, their results showed that MetAP‐2 was tethered to SCF complex (β‐TRCP) and ubiquitinated for degradation.^2^ Soon later, this group continued to employ this concept to design chimeric molecules to target ER and AR.^25^ They synthesized a 10‐aa IκB‐α peptide covalently linked to estradiol (E2) or dihydroxytestosterone (DHT) and confirmed both hybrid molecules functioned in vitro and in vivo in cells.^25^ These pioneer studies started the era for the peptide‐based PROTAC technology (Figure 3).

After the studies on ER and AR, Montrose and his colleagues used peptide‐based PROTAC to target the cancer‐forming X‐protein from HBV.^26^ The X‐protein is essential for viral replication, with 154 aa residues, and is a major risk for patients with hepatocellular carcinoma (HCC) developed from chronical infection with HBV. They intended to induce a poly‐arginine cell‐penetrating peptide (CPP) so that the PROTAC is cell‐permeable. They provided evidence that the homo peptide‐based PROTAC destroyed the X‐protein in HepG2 cells effectively.^26^

Perseveringly, Crews group designed PROTACs using FKBP12 ligand and dihydrotestosterone to target FKBP12 and AR in a model cell.^27^ They proved that this PROTAC‐mediated protein degradation could be a general strategy to deplete proteins, which they called “chemical knockouts” of proteins.^27^ At the same time, Kim group took advantage of the interaction between pVHL (von Hippel‐Lindau) and HIF‐1α (hypoxia‐inducible factor 1α), and designed a PROTAC based on a peptide from HIF‐1α.^28^, ^29^ They synthesized estradiol‐HIF‐1α octapeptide (Met‐Leu‐Ala‐Pro^OH^‐Tyr‐Ile‐Pro‐Met) to successfully target ER in living cells.^28^, ^29^ They subsequently confirmed that this PROTAC targeted ER and was able to inhibit the differentiation of endothelial cells in a three‐dimensional angiogenic sprouting assay.^30^ Kim group claimed their first report on the PROTAC that is permeable to cells since Crews group used microinjection to deliver the primary PROTACs.^2^, ^25^ The same year, Crews group searched for seven amino acids from HIF1‐α that recognizes VHL, aimed to overcome the obstacle of membrane permeability.^27^ For this purpose, a poly‐D‐arginine tag derived from HIV TAT was used to merge to the carboxyl terminus of the peptide to allow the hybrid macromolecule to confer cell permeability and prevent nonspecific proteolysis.^27^ Interestingly, Kim group extended their study into using apigenin, which is a low estrogenic flavonoid phytochemical found in some special diets with anticancer features.^31^ Their design consisted of apigenin, a linker, and an E3 ubiquitin ligase recognition motif (H2N‐Leu‐La‐Pro^OH^‐Tyr‐Il2‐OBn). They demonstrated that this apigenin‐based PROTAC effectively degraded aryl hydrocarbon receptor (AHR) in living cells.^31^, ^32^

Beside the usage of E3 ubiquitin ligase for ubiquitin‐mediated degradation by proteasome, Bauer et al subsequently adopted chaperone‐mediated autophage (CMA), by synthesis of a pentapeptide (KFERQ) to link two different HSP70 binding motifs to direct mutant huntingtin protein for degradation.^33^ Later on, Fan et al tried to recruit autophage system by a full peptide for the protein degradation.^34^ They took advantage of CMA and designed PROTACs against death associated protein kinase 1 (DAPK1), scaffolding protein PSD‐95, and a‐synuclein. Their design included a pentapeptide CMA‐targeting motif that recognizes autophage system, a linker containing cell membrane–penetrating domain (CMPD), and a peptide for recognition of targeted proteins. They confirmed that this homo multiple‐peptide efficiently knocked down the targeted protein not only in the cultured cells but also in the brains of intact rats because of CMPD, which made the peptide permeable to plasma membrane and the blood brain barrier.^34^

Obviously, these initial PROTAC technologies were based on the short peptide sequence to recognize an E3 ubiquitin ligase. Therefore, researchers named this PROTAC peptide‐based PROTAC.^6^, ^35^ To date, different peptides were examined to recruit E3 ubiquitin ligases including SCF complex, VHL, and CMA. As a proof of concept, the peptide‐based PROTACs proved that induction of the targeted protein degradation is a potent way to inhibit the activity of the targeted proteins. However, the problem for these peptide‐based PROTCs was due to their difficulty to permeate the cell membrane. That is the reason why Crews group initially used microinjection to deliver the PROTAC into living cells.^2^, ^25^ Sooner, Crews group used HIV tag to fuse the peptide and could be able to allow the PROTAC being transferred into the cell,^27^ and Kim group directly synthesized a cell‐permeable PROTAC.^28^, ^29^ Afterwards, the peptide‐based PROTACs always recruited CPPs.^26^, ^33^, ^36^

To show the biological function of the peptide‐based PROTACs, Crews group examined their PROTACs on targeting ER (named Protac‐B for ERα) and AR (named Protac‐A for AR). Intriguingly, the designed two PROTACs demonstrated a great accuracy to AR and ER, as both Protac‐A and Protac‐B did not affect the proliferation of cells lacking ERα and AR.^37^ Tang et al later on demonstrated that the DHT‐PROTAC promoted AR degradation in LNCaP cells, confirmed the role of the PROTAC on ERα or AR positive cells.^38^ They investigated the degradation of AR for the effect on cell proliferation and viability for prostate cancer cells sensitive to androgen. As expected, the DHT‐PROTAC worked specifically on the androgen positive cells.^38^ The peptide‐based PROTAC against ERα was further designed effectively in a MCF‐7 mouse xenograft model.^39^

However, the activity of these peptide‐based PROTACs was low and remained at the micromolar range. The main obstacle may be the poor cell permeability. It seems that the homo peptide based PROTACs, for instance PROTACs targeting Tau, were able to transport into the cell membrane because the addition of CPP (D‐Arg_8‐9_).^36^, ^40^ Another problem for these peptide‐based PROTACs is the size of the chimeric molecule, which could be recognized by immune system to produce antibodies. This may damper the clinical applications in human as the produced antibodies may neutralize the effect of the molecule in vivo. Fortunately, continual attempts on the improvement of the peptide‐based PROTACs have promoted the development of a new generation of PROTACs.

## 4 SMALL MOLECULE‐BASED PROTAC

**Figure 4:**
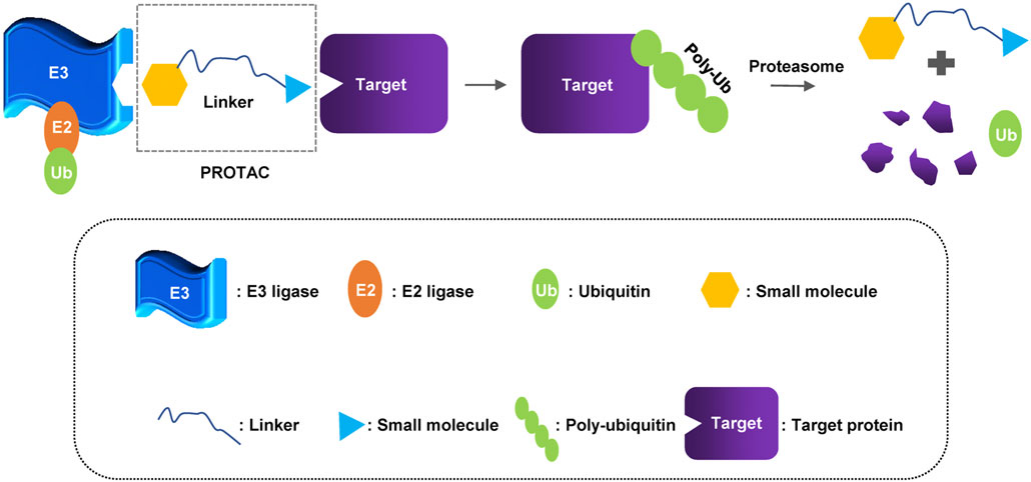
A schematic diagram of the small molecule‐based PROTACs. This proteolysis targeting chimera (PROTAC) consists of a ligand on an E3 ubiquitin ligase, a linker, and a ligand on targeted protein

The peptide‐based PROTAC takes advantage of a specific peptide on the reorganization of a specific E3 ubiquitin ligase. The peptide is called a moiety of E3 ubiquitin ligase. It immediately draws the attention that a small molecule could be used as a moiety for recognizing an E3 ubiquitin ligase. Using small molecules as moiety of an E3 ubiquitin ligase led to the development of small molecule–based PROTAC (Figure 4).^35^

Small molecule‐based PROTAC has many advantages over peptide‐based PROTAC.^11^ Most importantly, a small molecule–based PROTAC has more potential of being developed into a drug because a small molecule is easier for human body to absorb than a peptide. Crews group turned their attention to generate this new generation of PROTAC technology. They were the first to link a nonsteroidal AR ligand (called selective AR modulator, SARM) to nutlin (a MDM2 ligand) by a Polyethylene Glycol (PEG)‐based linker.^41^ The MDM2 ligand nutlin is a set of compounds of imidazoline derivatives, which bound to MDM2 to block the interaction of MDM2 with p53. The synthesized SARM‐nutlin PROTAC was shown to induce strong degradation of AR in HeLa cells^41^ and in LNCaP cells.^38^

Considering using hydropholic tags (HyTs) to make the binding protein denatured for degradation, several HyTs were synthesized to examine their effects on different Halo tag fusion proteins. This HyTs proved a concept that small a molecule may bind to a protein and makes the protein in a denatured state, which is then degraded by ubiquitin proteasome or autophage.^42^ Simultaneously, Crews group replaced the HIF1α peptide with a small‐molecule ligand, the hydroxyproline moiety, which retains a high affinity and is critical for VHL binding.^43^ They synthesized PROTACs against ERRα, by incorporating a thiazolidndione‐based ligand specifically binding to ERRα into the hydroxyproline moiety (selected one from five hybrid molecules). They next synthesized a PROTAC against the serine‐threonine kinase RIPK2, by using the inhibitor vandetanib and the hydroxyproline moiety with a 12‐atom linker. They assessed the PROTACs on the expression of ERRα and RIPK2 in MCF‐7 breast cancer cells and human THP‐1 monocytes and proved that one PROTAC molecule could be able to mediate the degradation of multiple molecules of RIPK2 via ubiquitin‐proteasomal pathway.^43^ Furthermore, this hydroxyproline derivatives were further used for the synthesis of HaloPROTACs to target HaloTag7 fusion proteins, by developing chloroalkane‐containing PROTACs against Halo Tag7 fusion protein using the acyl amine moiety for recognizing VHL.^44^

Many of the small molecule–based PROTACs have been developed intensively to target the BET family proteins. BRD4 inhibitors have been extendedly studied and shown promises in anticancer therapy against MYC‐driven malignancies. The first effort was to link BET inhibitor JQ1 to a moiety of VHL.^45^ The designed PROTAC named MZ1 dramatically induced degradation of BRD4.^45^ In another test, BRD4 inhibitors were used to design a PROTAC named ARV‐825, which links a BRD4 binding moiety of triazolo‐diazepine acetamide class (OTX015) to pomalidomide, a cereblon binding moiety with a flexible polyethyleneglycol linker, to recruit the E3 ubiquitin ligase cereblon.^1^ Pomalidomide is a potent third‐generation immunomodulatory drug (IMiD) to induce degradation of essential Ikaros (IKZF1) transcription factors by interacting with the E3 ubiquitin ligase cereblon in multiple myeloma. Therefore, this design of PROTAC took advantage of small molecule as a moiety to recognize E3 ubiquitin ligase. Pomalidomide is another small molecule for the induction of E3 ubiquitin ligase used for the PROTAC design.^1^ The pomalidomide‐based PROTAC ARV‐825 was proved to function on different immune cells^46^ and greatly induced apoptosis in tumour cell line.^47^ Almost at the same time, Winter et al used the phthalimide as a moiety to hijack the cereblon E3 ubiquitin ligase to degrade BET family proteins.^48^ They used their selected direct‐acting inhibitor of BET bromodomains JQ1, through its carboxyl group and the aryl ring of thalidomide, to form a bifunctional hybrid molecule PROTAC. This phthalimide‐based design may have a great advantage for its application of clinics as phthalimide is an approved drug. Indeed, the designed PROTAC functions in vitro and in vivo in a leukaemia model.^48^

The small molecule–based PROTAC was further extended to the design of a PROTAC against oncogenic kinase BCR‐ABL.^49^ Inhibitors including imatinib, bosutinib, and dasatinib were linked to VHL E3 ubiquitin ligase ligand or pomalidomide (to recruit cereblon E3 ubiquitin ligase).^49^ For targeting kinases, a PROTAC against CDK9 was designed by using CDK9 inhibitor and thalidomide for targeting cereblon.^50^ To date, small molecule–based PROTACs have been generated to recruit MDM2, cellular inhibitor of apoptosis protein 1 (cIAP1), CRBN (cereblon), and, of course, VHL (for review, see Toure and Crews^51^).^15^, ^52^

To overcome the shortage of insufficient membrane permeability and stability of the peptide‐based PROTACs, Hashimoto group focused on using cIAP1, which promotes ubiquitination and proteasomal degradation of interacting proteins.^53^, ^54^, ^55^ They recruited a class of bestatin ester analogues (MeBS, methyl bestatin), a ligand binding to the baculoviral IAP repeat domains of cIAP1, to all‐trans retinoid acid to target CRABP‐I and II (cellular retinoic acid binding proteins‐I and II).^55^ Thus, the cIAP‐1‐based PROTAC could be able to induce the ubiquitination and degradation of the intracellular CRABP‐I/II proteins. Other cIAP1‐based PROTACs were designed to cross‐link inhibitor bestatin to small molecules of multiple targets, including Retinoic Acid Receptor (RAR), ER, AR, and TACC3.^54^, ^56^ However, because bestatin itself is not highly selective and lacks activity, the activity of multiple reagents of PROTAC is not high enough, and no candidate has entered animal experiments. Interestingly, Naito and Hashimoto named their designs SNIPER (Specific and Nongenetic IAPs‐dependent Protein ERaser).^57^ They kept SNIPER for their following studies on designing different hybrid molecules to target different proteins.^58^, ^59^, ^60^, ^61^, ^62^, ^63^, ^64^, ^65^ Unexpectedly, they deciphered that one of their PROTAC based on bestatin did not recruit cIAP‐1 but instead APC/C^CDH1^ complex.^59^

In summary, to date, different sets of small molecules have been developed as the moiety of E3 ubiquitin ligases including SCF, VHL, cereblon, MDM2, APC/C, and cIAP1.^35^, ^51^, ^56^ For the limited space in this review, the discovery of the small molecules as the moiety of E3 ubiquitin ligases could not be able to descript in this review.

## 5 TARGETING DIFFERENT PROTEINS FOR ANTICANCER DRUG DEVELOPMENT

**Figure 5:**
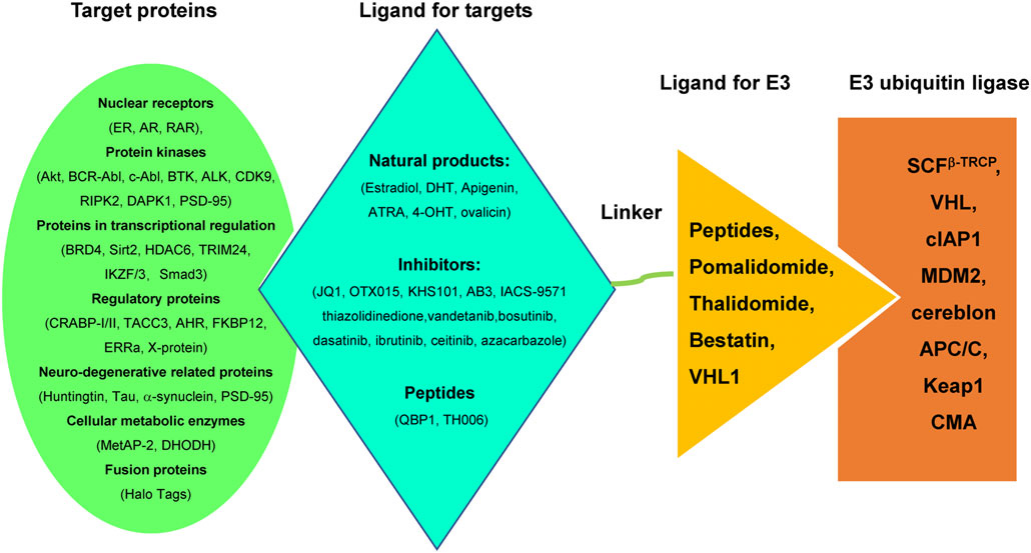
A summary of targeted proteins, ligands for target, ligand for E3 ubiquitin ligases, and recruited E3 ubiquitin ligases. MetAP‐2, methionine aminopeptidase‐2; ER, estrogen receptor; AR, androgen receptor; HTT, huntingtin protein; ERRα, estrogen‐related receptor alpha; AHR, activation of the aryl hydrocarbon receptor; CRABP‐I/II, cellular retinoic acid binding protein‐I/II; BRD4, bromodomain‐containing protein 4; TACC3, transforming acidic coiled‐coil‐3, spindle‐regulatory protein; DHODH, dihydroorotate dehydrogenase; DAPK, death‐associated protein kinase 1; PSD‐95, postsynaptic density protein 95; ALK, anaplastic lymphoma kinase; TBK1, TANK‐binding kinase 1; RIPK2, receptor‐interacting protein kinase 2; c‐Abl, Abelson nonreceptor tyrosine kinase; VHL, von‐Hippel‐Lindau ubiquitin ligase; CMA, chaperon‐meditated autophage; SCFb‐TRCP, Skip‐Cullin‐F box (β‐TRCP) ubiquitin complex; b‐TRCP, b‐transducing repeat‐containing protein; cIAP1, cellular inhibitor of apoptosis protein 1; MDM2, mouse double minute 2 homologue; APC/C, anaphase‐promoting complex/cyclosome

To date, more than 30 proteins critical for the development of diseases were targeted, with a major effort on the proteins for cancer therapy^6^, ^7^, ^18^, ^35^, ^56^ (Figure 5). The targeted proteins include nuclear receptors (ER, AR, and RAR), protein kinases (Akt, BCR‐Abl, c‐Abl, BTK, anaplastic lymphoma kinase [ALK], CDK9, RIPK2, DAPK1, and PSD‐95), proteins in transcriptional regulation (BRD4, Sirt2, HDAC6, TRIM24, IKZH1/3, and Smad3), regulatory proteins (CRABP‐I/II, TACC3, AHR, FKBP12, ERRα, and X‐protein), neuro‐degenerative related proteins (Huntingtin, Tau, a‐synuclein, and PSD‐95), cellular metabolic enzymes (MetAP‐2 and DHODH), and fusion proteins (Halo Tags).

### 5.1 Targeting nuclear receptors

Both peptide‐based and small molecule–based PROTACs were designed to target ER, AR, and later on RAR, which pioneered the field.^2^, ^25^, ^29^, ^54^ We have described the detailed designs of the PROTACs against ER and AR in the above sections.^25^, ^27^, ^28^, ^29^, ^37^, ^41^ Here, we intend to address some promising progress on the development of the PROTACa against ER or AR. One progress was to use 4‐hydroxy tamoxifen (4‐OHT) to link to methyl bestatin.^58^ Although named SNIPER, the hybrid molecule, SNIPER (ER)‐3, recruited cIAP1 E3 ubiquitin ligase to target ERα, and effectively induced the degradation of ERα. Consequently, SNIPER (ER)‐3 blocked the expression of PS2, a gene downstream estradiol, strongly induced the ROS production, and eventually led to necrotic cell death in MCF‐7 cells, an ER positive cell line, but not in other ER negative cells.^58^ Recently, a new PROTAC used nonsteroid selective ER degrader (SERD) was developed to generate more powerful and shorter active molecules to induce the degradation of ER.^66^

On the PROTACs against AR, Crews group used enzalutamide to optimally link to a VHL ligand and named the hybrid molecule ARCC‐4.^67^ They showed that ARCC‐4 induced the degradation of AR in not only all prostate cancer cell lines (VCaP, LNCaP, and 22Rv1) but also a breast cancer cell line (T47D). They further showed that ARCC‐4 inhibited androgen‐induced Prostate Specific Antigen (PSA) expression and apoptosis in VCaP cells. Intriguingly, they found that ARCC‐4 induced the degradation of AR mutants including F876L, H874Y, M896V, T877A, and L702H.^67^ Their studies based on cells provided hopes to cure AR mutant prostate cancers. Interestingly, Raina and his colleagues demonstrated that ARV‐771, a small molecule–based PROTAC using pan‐BET inhibitors suppressed both AR protein level and AR signalling, dramatically improved the efficacy in cellular models of castrate resistance prostate cancer (CRPC).^68^

### 5.2 Targeting transcriptional regulators bet family proteins

Crews groups tried to target BRD4, a bromodomain and extraterminal domain (BET) family member.^1^ They named their design ARV‐825, which recruits BRD4 inhibitor OTX015 connecting to pomalidominde, an E3 ligase cereblon binding moiety.^1^ The authors confirmed that ARV‐825 mediated a fast, efficient, and prolonged degradation of BRD4 as examined in all cell lines. Eventually, ARV‐825, by targeting BRD4, showed more effective inhibition of c‐MYC levels. This new strategy overcomes the problems of BRD4 inhibitions, which led to robust BRD4 protein accumulation.^1^ Later on in 2016, this group confirmed the effect of ARV‐825 in five MM cell lines [SKO‐007(J3), U266, RPMI‐8226, ARP‐1, JJN3] and an MM patient‐arised CD138+ MM cells. They showed that ARV‐825 was better than BET bromodomain inhibitors (BETi) (JQ1 and I‐BET151).^46^ The effect of ARV‐825 was further investigated to induce more apoptosis in CD34+ post‐MPN sAML cells.^47^ Specifically, ARV‐825 treatment led to robust and sustained depletion of BRD4 downstream genes including c‐Myc, CDK4/6, JAK2, pSTAT3/5, PIM1, and BclxL, but stronger increases of the levels of p21 and p27.^47^ These results suggested that PROTAC against BRD4 functions much better and the inhibitor of BRD4.

Slightly differently, Zengerle et al tethered JQ1, another inhibitor for BET family proteins, to a ligand for VHL. They observed that this PROTAC triggered in the intracellular destruction of BET proteins, preferably to BRD4. Intriguingly, the PROTAC not only led to a rapid, effective, and prolonged degradation of the BET family proteins but also caused the change of MYC, p21, and AREG, downstream of BRD4.^45^ Since BET proteins are critical for the expression of NF‐kB activated genes, a group reported that PROTACs against BET proteins dampened the pro‐inflammatory response in microglia after Lipopolysaccharide (LPS) challenge.^69^

Raina and his colleagues demonstrated that ARV‐771, another small molecule–based PROTAC using pan‐BET inhibitors, dramatically improved efficacy in cellular models of CRPC as compared with BET inhibition.^68^ Interestingly, ARV‐771 suppressed both AR protein level and AR signalling. This study provided evidence that a small molecule–based PROTAC functions in a solid‐tumour malignancy of CRPC.^68^ Further studies showed that PROTAC ARV‐771 treatment reduced leukaemia burden and improved survival of HEL92.1.7 cells‐engrafted NSG mice, better than the effect from OTX015.^47^

The effects of PROTACs‐based on BETi, ARV‐825, and ARV‐771 were recently examined in MCL cells. The results showed that BET‐PROTACs induced more apoptosis than BETi for MCL cells. Those BET‐PROTACs could be able to induce apoptosis for the ibrutinib resistant cells. The authors showed that BET‐PROTAC treatment decreased the mRNA and protein expressions more dramatically than BETi, for c‐Myc, CDK4, cyclin D1, and the NF‐kB transcriptional targets Bcl‐xL, XIAP, and BTK. Interestingly, BET‐PROTAC treatment induced the expression of HEXIM1, NOXA, and CDKN1A/p21. They finally declaimed that ARV‐771 possessed superior pharmacological properties compared with ARV‐825. Treatment with ARV‐771 significantly inhibited the in vivo tumour growth and improved the survival of MCL cell engrafted nude mice, compared to OTX015. Finally, those authors demonstrated that cotreatment of ARV‐771 with other drugs including ibrutinib, venetoclax (a BCL2‐antagonist), and palbociclib (a CDK4/6 inhibitor) had a synergistical effect on the induced apoptosis of MCL cells.^70^ Consistent with the about work, Qin later on discovered QCA570 as a potent PROTAC against BET proteins.^71^ More excitingly, Zhou et al designed a new PROTAC against BET family proteins and obtained a compound with 30pM concentration for effectively degrading BRD4.^72^

It seems that targeting BET family proteins using PROTACs becomes a hotspot recently. In 2018, Chong Qin designed a PROTAC using Oxazepines, a new class of BET inhibitors.^3^ This PROTAC named QCA570 was shown to effectively induce degradation of BET proteins and inhibited human acute leukaemia cell proliferation at low picomolar concentrations. They further demonstrated that QCA570 could completely abolish tumour growth in leukaemia xenograft models in mice.^3^ Recently, Zhang and his colleagues pursued PROTACs against BRD4 and other BET family members for preclinical studies.^73^ They found that the designed PROTACs strongly reduced the viability of myeloma cells and the effect was in a time‐dependent and concentration‐dependent manner. The myeloma cells after PROTAC treatment showed G0/G1 arrest, reduced expressions of CDK 4/6, increased expression of p21, and induction of apoptosis. The group reported that their PROTACs specifically decreased BRD4 downstream genes, including c‐MYC and N‐MYC. Notably, they showed that PROTACs overcame the drug resistance from bortezomib, dexamethasone, lenalidomide, and pomalidomide.^73^ They finally showed that PROTACs were able to induce a rapid loss of viability of primary cells from myeloma patients and inhibited the growth of MM1.S‐based xenografts in mouse.^73^ The PROTACs against BRD4 could also be improved by modification of hydroxylation of proline, which resulted in a PROTAC with over 100‐fold activity compared with conventional one.^74^ However, off‐targets were reported recently.^75^ A recent study extended designs of PROTACs against TRIM24, another bromodomain‐containing transcriptional regulator.^76^ This again encouraged to search for new path to undruggable targets.

### 5.3 Targeting protein kinases

Other proteins other than the BET family proteins have been also targeted by PROTACs. In 2016, a PROTAC was designed to target Akt using protein catalysed capture (PCC) agents to target a cell‐penetrating enzyme (Botulinum Neurotoxin Serotype A). They conjugated the PCC agent to a cell penetrating peptide HIV TAT peptide to allow an effective intake by cells. They further inserted two PEG spacers on both sides of a protected‐lysine residue. Basically, this PROTAC used 7 aa from HIF‐1α degradation peptide, ALAPYIP. This PROTAC was shown to promote the rapid degradation of Akt in live cancer cells.^77^

Next, Lai designed a PROTAC to target c‐ABL and BCR‐ABL by recruiting either cereblon or Von Hippel Lindau E3 ubiquitin ligases. They used inhibitors imatina, bosutinib, and dasatinb. During their study, Lai optimized the PROTAC development and proposed that both the target ligand and the recruited E3 ubiquitin ligase should be varied.^49^ In 2017, Robb successfully targeted CDK9, a ubiquitously expressed kinase that contributes to a variety of malignancies. This PROTAC used cereblon (CRBN) to mediate proteasomal degradation of CDK9. The authors examined this PROTAC in HCT116 cells and observed that it selectively degrades CDK9 without affecting other CDK family members.^50^ More PROTACs on CDK9 were developed by using a natural product Wogonin, which is similar to CDK9 inhibitor Flavopiridol.^78^

In 2018, Zhang reported their design of a PROTAC against ALK by using ALK inhibitors. These PROTACs against ALK were named MS4077 and MS4078. They showed that the PROTACs significantly decreased cellular levels of ALK fusion proteins in different cell lines including SU‐DHL‐1 (lymphoma) and NCI‐H2228 (lung cancer).^79^ Another group reported their design on ALK PROTAC using small molecule as ligand to connect E3 ubiquitin ligase.^80^ Kang et al later on proved that a synthesized PROTACs against ALK (based on VHL) worked in vivo.^81^ It seemed that the PROTACs are good for mouse pharmacokinetic study for in vivo efficacy test.^73^ The designed PROTAC against ALL also promoted the degradation of other kinase such as PTK2, Aurora A, FER, and RPS6KA1.^80^

To date, PROTACs targeting RAR,^54^ PI3K,^82^ CRABPI/II,^53^, ^55^ ALK4,^83^ Smad3,^84^ CDK9,^50^, ^85^ HDAC6,^86^ Sirt2,^87^ BTK,^88^, ^89^, ^90^ CK2 casein kinase 2,^91^ and TBK1^92^ are also reported.^93^ Most of the proteins are cellular located or nuclear located. However, for the receptors such as tyrosine kinase receptors (EGFR), it remains to question whether a PROTAC works or not. To examine this possibility, Crews group conjugated an EGFR binding element (Iapatinib) to a ligand of VHL for targeting EGFR, HER2, and c‐Met.^94^ Interestingly, the PROTAC mediated the internalization of EGFR and sorted to lysosomal degradation,^94^ although the RTKs usually prefer to internalize into a recycle endosome.^95^

## 6 REMARKS

Although it is very promising to use PROTAC for drug development, it remains of many concerns about the clinical application. These concerns include the off‐target, cellular permeability, stability, and large molecular weight. Another problem is the difficulty of synthesis of the hybrid molecule, including optimizing the linker length and composition. The good news is that many groups started to overcome these problems by different ways.^7^, ^11^, ^35^, ^96^ A new strategy for shortening the PROTAC, click‐formed PROTAC (TCLIPTAC), is to separate the macrohybrid molecule into two parts, a tetrazine tagged ligand for target and a trans‐cyclo‐octene tagged ligand for E3, which are able to be “clicked” together in the cells to form a PROTAC.^97^ This click reaction also provides an easier way to the synthesis of PROTACs in vitro.^98^

A plausible feature for the PROTAC technology is its potential for development of drugs on the undruggable proteins.^14^, ^93^ However, the current successful PROTACs still largely used small molecules to target the druggable proteins with their inhibitors or ligands. This is mainly because the small molecules have good features of binding the targeted proteins. To date, it remains of an obstacle for the discovery of small molecule moiety to different targets. One direction is to find a peptide epitope based on protein‐protein interaction. This will open a broad way for the discovery of new drugs.

## CONFLICTS OF INTEREST

There are no other conflicts of interest to disclose.
